# Methaemoglobinaemia as an Alternative Diagnosis for Shortness of Breath: A Case Report

**DOI:** 10.7759/cureus.83525

**Published:** 2025-05-05

**Authors:** Thiri Thiri, Su Su San, Zaw Thant Lwin

**Affiliations:** 1 Clinical Neurophysiology, Calderdale Royal Hospital, Halifax, GBR; 2 General Medicine, King's Mill Hospital, Sutton-in-Ashfield, GBR; 3 Hematology, King's Mill Hospital, Sutton-in-Ashfield, GBR; 4 Acute Medicine, Royal Derby Hospital, Derby, GBR

**Keywords:** acute care medicine, cyanosis, dapsone, dyspnoea, methaemoglobin

## Abstract

Methaemoglobinaemia is rarely considered as a differential diagnosis in patients presenting with shortness of breath and cyanosis. It can occur due to either congenital or acquired causes. The effect of medication is an important consideration as an acquired cause of methaemoglobinaemia, and if the diagnosis is missed, it may result in a fatal outcome. This scenario highlights the importance of awareness through thorough history-taking and careful review of investigations. Dapsone (4,4’-diaminodiphenyl sulfone) was identified as the main contributing factor in this case, and the symptoms resolved following its immediate discontinuation, with an improvement in oxygen saturation and a reduction in methaemoglobin levels.

## Introduction

Methaemoglobin (MetHb) is a naturally occurring oxidised metabolite of haemoglobin, and physiological levels (<1%) are considered normal [1]. A MetHb level of <1% is maintained steadily by red blood cells through enzymatic pathways [2]. Problems arise when MetHb levels increase, resulting in reduced oxygen-binding capacity and tissue hypoxia once levels exceed 3% [1]. This can occur if congenital or acquired causes, including exposure to oxidising agents, disrupt the enzymatic pathways. Certain medications act as oxidising agents that can induce methaemoglobinaemia. Among these, dapsone (4,4’-diaminodiphenyl sulfone) is known to be the most common cause. Clinical symptoms vary, ranging from being asymptomatic to confusion, cardiovascular collapse, and ultimately death, depending on the serum concentration of MetHb [2,3]. However, the level of MetHb may not always correlate with the clinical symptoms. Therefore, awareness of methaemoglobinaemia in patients presenting with cyanosis is important [3]. When MetHb levels reach 10% or more, the clinical symptom of cyanosis may be observed [1].

In this patient’s case, she was clinically stable apart from symptoms of cyanosis, tiredness, and shortness of breath with low oxygen saturation, making diagnosis a challenge. After a thorough review of her blood investigations and consideration of rare causes related to her clinical presentation, further enquiry into her drug history led to the correct diagnosis.

This article was previously presented as a poster at the Society for Acute Medicine’s 18th Annual International Conference on 10-11 October 2024.

## Case presentation

A 58-year-old lady presented to the hospital after noticing bluish discolouration of her lips, nail beds, and left mastectomy scar, accompanied by reduced SpO₂ of 88%. She also reported a one-day history of tiredness and mild shortness of breath. She denied experiencing chest pain, cough, haemoptysis, palpitations, syncope, or leg swelling. However, she reported right calf pain one week prior, although a Doppler ultrasound of the leg was normal. She was independently mobile at home.

Her comorbidities included hypertension, a hysterectomy for endometriosis in 2002, a laparoscopic cholecystectomy for gallstones in 2010, and a left mastectomy for breast cancer four months ago. There was no history of cardiovascular or respiratory disease. She was taking regular antihypertensive medication. Chemotherapy had commenced five days earlier under the care of her oncologist.

On examination, she was not in respiratory distress, but required 4 litres of oxygen via nasal cannula to maintain an oxygen saturation of >94%. Her lips and nail beds were mildly cyanotic, though there was no visible cyanosis of the tongue or buccal mucosa. The remainder of the systemic examination was unremarkable.

Admission venous blood gas (VBG) analysis showed a normal pH (Table 1). Full blood count revealed a normal haemoglobin level, raised white cell count with neutrophilia, and a normal platelet count (Table 1). C-reactive protein (CRP), clotting profile, liver function, and renal function tests were all within normal limits. D-dimer was elevated (Table 1), and the Wells' criteria for pulmonary embolism (PE) were 4. Chest X-ray and ECG revealed no abnormalities. COVID-19 swab was negative.

**Table 1:** Blood results on admission g/L: grams per litre, ng/ml: nanograms per millilitre

Test	Patient values	Reference range
pH	7.41	7.35-7.45
Haemoglobin	144 g/L	121-151 g/L
White cell count	15.8 x 10^9^ /L	4-11 x10^9 ^/L
Platelet	327 x 10^9 ^/L	150-450 x10^9 ^/L
D-dimer	868 ng/ml	≤500 ng/ml

Initially, PE was the primary differential diagnosis based on the patient’s clinical presentation of breathlessness, low oxygen saturation, and a moderate-risk Wells' criteria for PE, along with a raised D-dimer in the context of malignancy. A computed tomography pulmonary angiogram (CTPA) was therefore requested, and treatment-dose enoxaparin was commenced for suspected PE. However, as the patient’s main presentation was cyanosis, alternative causes of cyanosis, aside from low oxyhaemoglobin levels associated with PE, were considered.

In addition, the patient’s white cell count was found to be raised in the absence of symptoms or signs of infection, and her CRP was normal. Upon further enquiry into her drug history, the patient reported that she had started oral dexamethasone and dapsone 100 mg daily the day prior to admission. This raised the suspicion of methaemoglobinaemia. The VBG report showed a MetHb level of 11.2% (reference range: 0-1.5%). The raised white cell count could be attributed to the dexamethasone. Taking into account this new information regarding her medication history, along with a more detailed evaluation of her investigations, the working diagnosis shifted to methaemoglobinaemia, with PE remaining a differential diagnosis to be excluded.

Dapsone was discontinued, and TOXBASE was consulted. The patient did not require methylene blue and improved with supplemental oxygen delivered via nasal cannula. An arterial blood gas (ABG) performed during the medical team review revealed an SaO₂ of 94%, while SpO₂ was 88% on room air. The MetHb level had decreased to 9.8%. The oncologist was updated regarding the clinical presentation and the discontinuation of dapsone. Typically, co-trimoxazole is prescribed for *Pneumocystis pneumonia* prophylaxis in chemotherapy patients; however, due to a history of urticaria with trimethoprim, dapsone had been used instead. The oncologist agreed with the decision to withhold dapsone.

**Figure 1:**
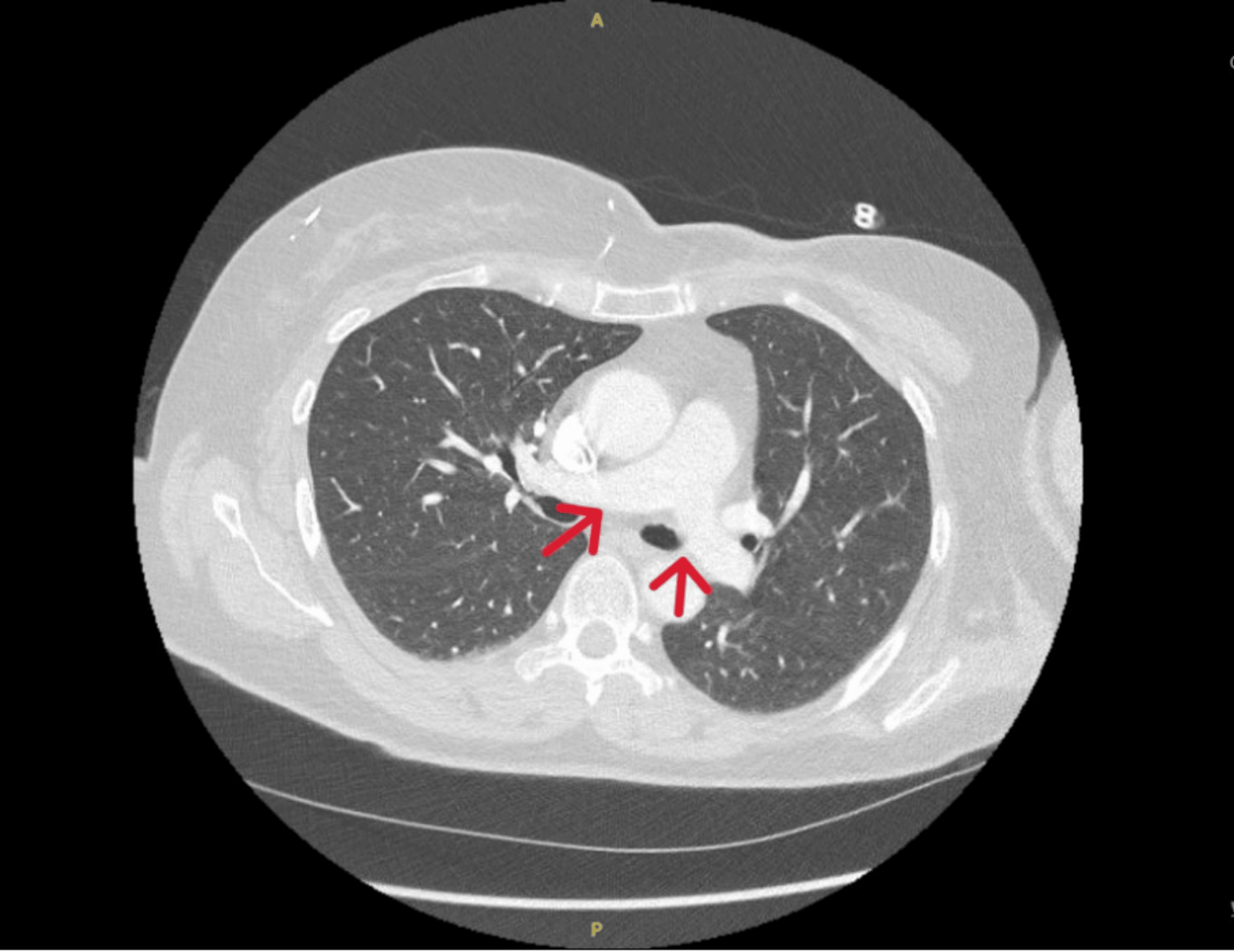
CTPA excluding the possibility of PE CTPA: computed tomography pulmonary angiogram, PE: pulmonary embolism

The CTPA reassuringly excluded PE (Figure 1), and the patient was subsequently discharged with follow-up under the oncology team. A VBG taken at the oncology clinic showed a normal MetHb level of 1% one week later. Her symptoms had completely resolved at follow-up.

## Discussion

Dapsone is a sulfone antibiotic. When metabolised in the liver via the cytochrome P450 pathway, dapsone produces potent oxidants that cause haemolytic anaemia and methaemoglobinaemia [2]. It has a dual function, exerting antimicrobial/antiprotozoal and potent anti-inflammatory effects by inhibiting folate synthesis [1,2]. Dapsone is traditionally used in the treatment of leprosy and certain dermatological conditions. Additionally, one of its off-label uses is for the prophylaxis of *Pneumocystis jiroveci* pneumonia [2]. It can be administered orally, topically, or via injection. Orally administered dapsone is slowly absorbed, reaching peak plasma concentration after approximately four hours. Its half-life is 1.1 hours, and elimination from the body occurs over approximately 30 hours [4].

Methaemoglobinaemia is physiologically present in the blood at levels of less than 1%. Two mechanisms maintain MetHb at a constant level by reducing MetHb to Hb: the cytochrome b5 reductase pathway and the nicotinamide adenine dinucleotide phosphate-dependent MetHb reductase pathway, which requires the cofactor methylene blue or riboflavin for activation [5]. Oxidation of haemoglobin iron from the ferrous form (Fe²⁺) to the ferric form (Fe³⁺) results in the formation of MetHb, which leads to reduced haemoglobin binding to oxygen (functional anaemia) and impaired oxygen loading at the tissue level [6-9]. MetHb shifts the oxygen-haemoglobin dissociation curve to the left, decreasing oxygen release and tissue hypoxia.

MetHb levels may rise due to congenital enzyme deficiencies or acquired exposure to exogenous oxidising agents such as drugs, chemicals, or toxins, including dapsone, local anaesthetic agents, and nitroglycerin. The incidence of dapsone-induced methaemoglobinaemia is approximately 3% among haematopoietic stem cell transplant recipients receiving dapsone for *Pneumocystis jirovecii* pneumonia prophylaxis [10].

Methaemoglobinaemia presents variably depending on MetHb levels. Cyanosis typically appears when levels exceed 10%. At levels between 20% and 50%, symptoms may include headache, dyspnoea, lightheadedness, weakness, palpitations, chest pain, and confusion. When MetHb levels rise to between 50% and 70%, cardiac arrhythmia, seizures, delirium, coma, and profound acidosis may occur. Death usually results when MetHb levels exceed 70%. Hypoxia and cyanosis (defined as the presence of more than 5 g/dL of deoxygenated haemoglobin) may also be seen in patients with underlying cardiac or pulmonary disease. Methaemoglobinaemia should be considered in cases of cyanosis unresponsive to high oxygen concentrations without a cardiac or pulmonary disorder.

Pulse oximetry measures only two types of haemoglobin. As MetHb levels rise, oxygen saturation readings on pulse oximetry fall. The presence of an alternative type of haemoglobin should be suspected when pulse oximetry oxygen saturation is lower than that obtained via ABG analysis [1]. The oxygen saturation gap is the difference between the oxyhaemoglobin concentration measured by pulse oximetry and the arterial oxygen concentration from a standard blood gas analyser. A gap greater than 5% suggests the presence of methaemoglobinaemia, sulfhaemoglobinaemia, or carboxyhaemoglobinaemia [11].

Methaemoglobinaemia can be diagnosed using various laboratory tests. For hereditary causes, haemoglobin electrophoresis and DNA sequencing of the globin chain gene can identify haemoglobin M, which leads to congenital methaemoglobinaemia. Specific enzyme assays are performed to identify inherited enzyme deficiencies. Co-oximetry and potassium cyanide tests are also used to confirm the presence of MetHb.

An arterial blood sample with a characteristic chocolate-brown colour may also aid in diagnosis. MetHb levels can be measured at the bedside using point-of-care blood gas analysers immediately after ABG collection [12].

Management is guided by TOXBASE guidelines for methaemoglobinaemia. Treatment options depend on MetHb concentration and the severity of symptoms. High-flow oxygen, administration of methylthioninium chloride, O-negative red blood cells, or exchange transfusion are recommended [13]. Removal or discontinuation of the oxidising agent is also a key component of treatment.

## Conclusions

Whilst common cardiorespiratory presentations are frequently encountered, it is important to keep an open mind to rarer differentials and pay close attention to investigative findings that may contradict the working diagnosis, such as a high MetHb level identified on a routine VBG, as seen in this case. Methaemoglobinaemia should be considered as a differential diagnosis in a cyanosed patient with a normal PaO₂ and an unexplained decrease in SpO₂, particularly in the context of a normal cardiopulmonary status. Physicians and other healthcare workers should always consider adverse medication reactions as part of the differential diagnosis in cases of atypical clinical presentation.

This case report highlights the importance of thorough history taking and careful review of investigations in reaching an accurate diagnosis. In this scenario, the unusual clinical presentation prompted consideration of alternative differential diagnoses, ultimately leading to the identification of this rare condition following a review of blood results and the relevant drug history, central themes of the case. In the future, early and accurate diagnosis in similar cases may help to avoid unnecessary radiation exposure and prolonged hospital stays.

## References

[1] Mahmood N, Khan MU, Haq IU, Jelani FA, Tariq A (2019). A case of dapsone induced methemoglobinemia. J Pharm Policy Pract.

[2] Burke P, Jahangir K, Kolber MR (2013). Dapsone-induced methemoglobinemia: case of the blue lady. Can Fam Physician.

[3] El-Husseini A, Azarov N (2010). Is threshold for treatment of methemoglobinemia the same for all? A case report and literature review. Am J Emerg Med.

[4] Hayama Y, Imanishi H, Yoshimoto N, Sugawara K, Tsuruta D (2020). A case of dapsone-induced mild methemoglobinemia with dyspnea and cyanosis. Acta Dermatovenerol Croat.

[5] Zuidema J, Hilbers-Modderman ES, Merkus FW (1986). Clinical pharmacokinetics of dapsone. Clin Pharmacokinet.

[6] Khanal R, Karmacharya P, Pathak R, Poudel DR, Ghimire S, Alweis R (2015). Do all patients with acquired methemoglobinemia need treatment? A lesson learnt. J Community Hosp Intern Med Perspect.

[7] Ash-Bernal R, Wise R, Wright SM (2004). Acquired methemoglobinemia: a retrospective series of 138 cases at 2 teaching hospitals. Medicine (Baltimore).

[8] Zosel A, Rychter K, Leikin JB (2007). Dapsone-induced methemoglobinemia: case report and literature review. Am J Ther.

[9] Mansouri A, Lurie AA (1993). Concise review: methemoglobinemia. Am J Hematol.

[10] Darling RC, Roughton FJ (1942). The effect of methemoglobin on the equilibrium between oxygen and hemoglobin. Am J Physiol.

[11] Sangiolo D, Storer B, Nash R (2005). Toxicity and efficacy of daily dapsone as Pneumocystis jiroveci prophylaxis after hematopoietic stem cell transplantation: a case-control study. Biol Blood Marrow Transplant.

[12] Skold A, Cosco DL, Klein R (2011). Methemoglobinemia: pathogenesis, diagnosis, and management. South Med J.

[13] (2025). The Primary Clinical Toxicology Database of the National Poisons Information Service. https://www.toxbase.org/information/treatment/methaemoglobinaemia-features-and-management/.

